# The autonomy principle in companion veterinary medicine: A critique

**DOI:** 10.3389/fvets.2022.953925

**Published:** 2022-09-30

**Authors:** Karen M. Hiestand

**Affiliations:** Centre for Mammal Communication and Cognition, School of Psychology, University of Sussex, Brighton, United Kingdom

**Keywords:** autonomy, veterinary ethics, owner autonomy, animal patient, animal welfare, best interests, informed consent

## Abstract

Following developments in human medical ethics, veterinary ethics has similarly shifted from a historic paternalistic approach, toward greater respect for autonomy. Veterinarians operate within a tripartite system where there is separation of doctor/patient dyad by animal owners. As such there are fundamental differences between veterinary and human medical sectors regarding application of the autonomy principle—specifically, to whom is autonomy afforded? This paper argues that the accepted transference of autonomy to owners constitutes a corruption of the principle. Privileges owners exercise over animal treatment decisions relate to their rights over property use, rather than application of self-rule over one's own person as described in bioethics literature. To highlight issues with the status quo, this paper outlines the negative consequences of “owner autonomy” on animal (patient) welfare, integrity of the veterinary profession's social contract and professional autonomy. A way forward is proposed that places greater emphasis on animal (patient) welfare being explicitly at the center of veterinary treatment decision-making *via* recognition that all such decisions are made by a proxy, and therefore more appropriate frameworks ought to be engaged, such as a best interests paradigm.

## Introduction

Broadly speaking, autonomy refers to one's ability to choose for oneself, or self-rule [(1), p. 101]. Philosophically, this is based on the idea that an individual can control their actions and is also best placed to choose these actions due to their unique perspective of what matters to them. It is conditional on an individual being able to formulate preferences and desires, as well as being in receipt of all pertinent information with the ability to reason and consider options. In medical ethics it has come to encompass a patient's right to choose their medical care, and self-determination over bodily integrity.

Both human and veterinary medicine historically operated under a “doctor knows best” paradigm, what is considered a paternalistic model (2, 3). The term paternalism has been with us since the 1880s and means governance “as by a father” and there are two aspects which are presupposed: first that a father acts beneficently on behalf of his children, and second, that he will make decisions for their welfare rather than them making decisions for themselves [(1), p. 215]. This principle has since been applied to the medical setting where the practitioner, through superior knowledge, training and experience assumes the position of “father” by overriding a patient's preferences with the goal of benefiting them or preventing them being harmed. Paternalism, despite modern, negative connotations, thus relies principally on beneficence, acting for the good of others, and non-maleficence, doing no harm.

The principle of respect for patient autonomy began to supplant paternalism in the medical sphere after World War II and the actions of Nazi doctors. The resultant Nuremberg Code highlighted the importance of voluntary consent over one's person. This concept was incorporated into principlistic ethical frameworks in the 1970s as respect for persons in the *Belmont Report* (4) and autonomy in Beauchamp and Childress's *Principles of Biomedical Ethics* [(1), originally published in 1979]. The latter authors work has been highly influential in the context and practicalities of medical ethics. The respect for autonomy principle is considered to have moral priority, particularly in western, more libertarian societies where an autonomy based on self- interest has become dominant in professional guidance in medicine (5, 6) assisted by a growing distrust of the medical profession (7). In contemporary healthcare law, autonomy can be viewed as the core legal principle (8) with doctors instructed to “respect a patient's decision to refuse an investigation or treatment, even if you think their decision is wrong or irrational… You must not …. put pressure on a patient to accept your advice” (9). However, despite the influence of the autonomy principle, acceptance of its primacy in medical ethics is not without debate (2, 7, 10–14).

The evolution of veterinary ethics tends to follow developments in the human medical field. An historic paternalistic approach in animal medicine has likewise migrated toward a greater respect for autonomy and the embedding of informed consent, though the shift may not have been as pronounced as that in the human medical field (8). However, as the veterinary sector generally operates under a complex tripartite system with separation of the doctor/patient dyad by an animal owner, it is pertinent to question: whose autonomy are vets respecting?

It is widely accepted in the sphere of veterinary medicine that the autonomy of an animal *owner* be respected (15–18). An explanation for this may be the more utilitarian nature of the human-animal relationship in the agricultural history of veterinary medicine, which led to animal interests in treatment decision making being rendered moot in favor of economic and production-based considerations (19). It is of course, possible to argue there is no fundamental difference in the moral status of animals used in production systems vs. those used for human companionship. For example, Wilkie (20) illustrated how human relationships with livestock animals are complex, context specific and changeable. E.g., can depend on animal “career path” (breeding animals vs. animals produced for slaughter), or when individual animals are recognized and transition to become “more than just an animal” [(20), p. 215]. However, as human—companion animal bonds have evolved to be more relational in nature [(16), p. 125–126] and greater moral value is placed on companion animals [(21, 22), p. 58] this paper addresses the issue specifically in the companion animal context. I will raise questions around the appropriateness of prioritizing respect for owner autonomy by highlighting how transposition of autonomy to owners corrupts the intent of the principle and go on to consider the implications for animal welfare, public trust in the veterinary profession and professional autonomy.

### Can animal patients be autonomous?

How is it that autonomy has been translated in veterinary medicine from a principle respecting a patient's right to make decisions about their own health care, to a respect for proxies (owners) making decisions about another sentient individual?

Theories of autonomy generally require two essential conditions—liberty (independence from controlling influences) and agency (capacity for intentional action), [(1), p. 102]. While some have argued that chimpanzees can satisfy both requirements necessary to be considered autonomous (23), and perhaps many non-human animals could according to a model set out by Beauchamp and Childress in 2019 (24), the liberty of animal patients is generally considered constrained by their legal status as property (8) and they remain legally object, rather than subject.

Animal agency is a more nuanced consideration. Our understanding of animals' ability to choose what happens to them has been utilized in animal welfare research through preference testing, which demonstrates that animals are able to express choices about their environment [see Dawkins (25) and Fraser and Nicol (26)]. Furthermore, in the companion animal context, there is a nascent interest in permission-based or cooperative care (27) where the aim is to afford animals greater agency to acquiesce or dissent to treatment. Despite these examples, companion animal agency in the form of dissent is usually responded to *via* the use of restraint (physical and chemical) or behavioral modification such as training, desensitization, and food rewards (28). This is comparable to the status of children in health care, where despite being legally subject, in possession of individual rights and perfectly able to dissent, they are similarly manipulated or forced to experience treatments such as being physically restrained for blood draw or vaccination (24). These examples illustrate that it is not whether animals (or children) can be autonomous, but whether we ought to respect their autonomous decisions which is at issue here. Similarly, Beauchamp and Wobber (23) advise that despite the abilities of chimpanzees to exercise autonomy, this does not impart a duty to respect it.

Autonomy, particularly when operationalised as consent, also relates to capacity. Capacity as described under the mental capacity act, is simply understood as being able to make a decision, and in human's this entails the following standards regarding information, which you must be able to do all of. One must understand and retain information, weigh it up and communicate a decision. Animal patients are not able to demonstrate capacity in this manner, and as such cannot give autonomous consent, which leads, alongside their lack of liberty and debated agency, to decisions about their care being necessarily made by a proxy or surrogate.

### Corruption of the autonomy principle

This paper argues that owner autonomy, including when operationalised as informed consent, is a corruption of the principle. Individual autonomy is concerned with the expression of a subject's self-determination over bodily integrity, and informed consent acts as a practical manifestation of the principle (29). As animals don't have capacity to consent, autonomy in the veterinary sector cannot be based on a patient's self-determination. The rights that owners exercise over animal treatment decisions is in regard to property use, rather than a respect for autonomous decision making for one's own body as the genesis and debate about the principle advocates. The idea that owners are exercising autonomy in companion animal healthcare decisions falls at the first hurdle by necessarily being a proxy-decision, made for another, rather than being about the self. As it currently stands, the autonomy principle in healthcare affords protection to human patient interests yet provides none to animal patients. While professional guidance states that veterinary surgeons must communicate effectively with owners and ensure informed consent is obtained before treatments or procedures are carried out (30), we ought to ask for what purpose; who is being protected, and why?

Informed consent in human medicine provides a framework for patients to protect their autonomous choices (18) and human doctors must seek patient consent before treatment. In contrast, informed consent in the veterinary setting affords the animal patient no such rights (31). The validity of informed consent in veterinary medicine is debated (8, 18, 32) with some suggesting it be removed altogether (19). Ashall et al., (18) postulate that informed consent in veterinary medicine exists to facilitate and articulate an owner's autonomous choices, to protect their emotional or economic interests regarding an animal, and not their own or the animal patient's bodily integrity. In the veterinary context, informed consent relates to animals' legal status whereby unconsented damage to property (animal) through medical treatment/surgery is an infringement of an owner's rights over that property under civil law (33–35).

Furthermore, obtaining consent has an additional purpose in providing legal protection for professionals [(36), however, see Passantino et al. (29)], with some defining its purpose as chiefly management of risk to the practitioner (37). While others call for more effective communication to increase overall validity (38, 39) the UK veterinary regulator highlights improved communication around consent specifically to reduce complaints (36). This somewhat mercenary application of informed consent in veterinary medicine highlights a significant mission drift from the original purpose of consent in human medical settings, to protect autonomous choice regarding one's body.

The role of autonomy and consent to afford protection to animal patient interests is entirely removed in the veterinary setting. Instead, both constructs are employed to protect the interests of owners and vets. It may be due to a lack of scrutiny that in non-human medicine we refer to proxy decisions made by owners as “autonomous” (8, 40). This may illustrate the pervasiveness of “animals as property” culture which creates an environment where such an assumption can go unquestioned, or a fundamental misunderstanding of the principle within the veterinary profession. Either way, the affordance of autonomy to animal owners deviates so fundamentally from its application in human medicine that the principle is rendered at best worthless in the veterinary sector, and at worst a dangerous corruption of the bioethical principle that threatens its very veracity.

### Implications of “owner autonomy”

What are the consequences for the vet profession of accepting that the principle of respect for autonomy be applied to the property rights of animal owners? This paper argues that not only can individual animal welfare be negatively affected by misapplication of the principle, but it may even have the power to undermine the position of the veterinary profession within society and harm vets themselves.

### Animal welfare

The veterinary professional duty to protect animal welfare is constrained both legally and regulatorily by affording primacy to the respect of owner autonomy. Despite Main's (41) advice that the veterinary obligation to animal welfare is greater than that for owner psychological wellbeing, a relatively commonplace scenario in companion animal practice is delayed euthanasia. This can occur when an animal's quality of life is such that the veterinarian believes euthanasia would be the best option, but an owner is emotionally unprepared to consent. Due to a plethora of affective reasons (fear of grief and loss, discomfort making life and death decisions etc.) the veterinary duty to animal welfare may be put aside in favor of considerations that prioritize the emotions and autonomy of an owner. Veterinarians regularly institutes a degree of palliative care until such time as an owner becomes emotionally prepared to end the animal's suffering, but despite the best therapeutic efforts, animals involved often experience negative welfare states. There is no single agreed way of measuring quality of life and opinions may differ as to the point where euthanasia is the best option, but I propose that most veterinarians have experienced scenarios when an animal suffered welfare harm while waiting for an owner to come to terms with the emotional impact of losing their animal companion. Harms to animals can be incurred through such futile, heroic or over treatment(s), where potential benefits are overemphasized, while pain and suffering are underestimated. These occasions conform to a therapeutic misconception (42) where there is a mistaken belief that individual decisions are made solely for the patients benefit (trying everything to save them), when other factors (e.g., emotional) are at play.

These welfare harms are accepted in part due to adherence to the legal requirement (afforded by animals as property) for owner autonomy in treatment decisions, and regulatory guidance for veterinary professionals. Veterinarians practicing in the UK declare an oath upon admittance to the Royal College of Veterinary Surgeons (RCVS) stating that they will “*…ABOVE ALL… ensure the health and welfare of animals committed to my care”* [(43), caps from original source] suggesting that beneficence toward patients takes precedence. RCVS guidance goes on to advise veterinary surgeons to “*make decisions on treatment regimens based first and foremost on animal health and welfare considerations*”, which seems to concur with the patient-first sentiment of the oath. However, the guidance immediately introduces the contradiction that vets “*also* (make decisions based on) *the needs and circumstances of the client*”. What are vets to do when the needs, circumstances and wants of an autonomous owner conflict with the welfare of the animal? Is it beholden upon the professional to intervene in the interests of benevolence on behalf of their patients?

Interventions which override owner autonomy are tricky to legally execute. Support for, and clarity on how and when a veterinarian ought to stage an intervention is lacking (8). RCVS provides us with the guidance that “*…veterinary surgeons and veterinary nurses must accept that their own preference for a certain course of action cannot override the owners' specific wishes, other than on exceptional welfare grounds*” (44). This raises the question: what is considered exceptional? One could argue that due to the commonality of futile treatments, such as delaying euthanasia, such cases represent “unexceptional negative welfare states”. Increasing rates of futile, heroic and over-treatments attest to the aforementioned therapeutic misconception, as patient welfare is easily lost in competition with desires of both owners, and sometimes vets, to “try everything”. Therapeutic interventions are rarely, if ever, pleasant for animals, with even seemingly benign treatments requiring transportation, handling, confinement, discomfort, and fear, all of which present welfare compromise to patients who cannot understand the purpose of their experience. Causes for increasing over-treatments are multiple, and include professional reasons (increased specialization, undergraduate training, desire for commercial and professional success) alongside increased owner demand for “gold standard” care. However, while some sections of the veterinary profession are increasingly debating the ethics of over-treatment (45, 46), countering owner desires remains challenging.

In cases where clear cruelty or neglect exist a veterinarian may have greater legal and moral justification to disregard an owner's wishes (15, 16, 41). However, Main [(41), p. 63] states “in most circumstances, a veterinary surgeon on his or her own has very limited powers without an owner's consent”. Convention dictates that despite there being instances in companion veterinary practice where arguably conditions of “unnecessary suffering” are met, considering these situations as offenses under the UK Animal Welfare Act (2006) very rarely occurs. In cases where an animal has suffered for days, weeks or months before presentation to a vet, be it a severe ear infection or a broken femur, it remains uncommon to be reported to authorities for investigation and prosecution under the Act. Owners are often protected from being viewed as having committed an offense if they seek veterinary treatment—no matter how delayed. Despite Deckha (47) suggesting that animals represent a “special type” of property in that they have added protections in legislation that other property types do not, the power of owner autonomy over animal property requires an extremely high bar of suffering—often far more than the animal protection legislation would suggest.

To resolve conflicted cases, veterinarians may exert benevolent influence less explicitly by subtle nudging or employing more “coercive or manipulative strategies” to encourage owners to make choices which best serve the patient [(15), see Yeates and Main (35) for a comprehensive list of the forms of influence veterinarians may exert]. Vets may tailor the options presented to owners, such as leaving out undesirable options or highlight those they feel are right. Employing language such as “you wouldn't want them to suffer” or appealing to justice principles; “they've had a good innings”, “it wouldn't be fair on them to keep going” may also be used. Situations where vets may morally exert varying degrees of influence over treatment decisions are discussed by Yeates and Main (35) who propose a test of “reasonableness” be applied in line with the existing legal duty of owners to ensure animals experience “reasonable” welfare (34, 48). Indeed, the British Veterinary Association advises veterinarians that “Promoting a patient's best interests sometimes requires ethically appropriate influencing of animal owners” [(49), p. 20].

Conflict between two “fundamental principles”; autonomy and beneficence, result in many dilemmas in bioethics and medical ethics (50) and represent a leading cause of work-related stress in vets (17, 51). While it is certainly common for both principles to converge such that a decision is both what an owner wants and what is best for the animal, it is when the principles diverge that difficulties arise. In these cases, one principle must take precedence at the expense of the other, but how ought we decide which matters more? Owner autonomy enjoys a legal standing *via* property rights that appears in practice to outweigh veterinary benevolence as supported by the veterinary oath and legal protection against animal harms. Beechum and Childress [(1), p. 101], state that autonomy ought not have dominance over other principles. They provide examples of situations when other principles should have precedence, including when public health may be endangered, resources are scare or there is potential harm to innocent others. It could be argued that animals are the innocents who might considered at risk of potential harm under this proviso. As such, if an owner's autonomous decision could bring potential harm to their animal, it can, even under the respect for autonomy principle, be acceptable and even imperative for a veterinarian to intervene. As presented, these interventions are difficult and hence uncommon, resulting in a status quo that allows owner autonomy to outweigh negative welfare in our most beloved animals.

### Veterinary social contract and public trust

Veterinarians, in common with other professions, maintain a contract with society whereby they are afforded privileges (the right to be called vets, practice veterinary medicine and gain financial reward for doing so) that are protected by law (in the UK, Veterinary Surgeons Act, 1966). In return, professions must perform a social good, utilize their privileges in a manner which adheres to the agreed ends of the profession, and serves the public interest. The public interest is determined by society and can alter over time as social ethics evolve. With companion animal work now accounting for ~ 70% of veterinary time (52), the focus has shifted to a profession serving a wider range of individual attitudes and desires, as opposed to the historical, more homogenous views of farmers.

Sentience and capacity for suffering increasingly affords a degree of moral status and duty of “moral respect” to animals [(53), p. 105–106] which constitutes a modern moral orthodoxy (54). The moral status of companion animals has evolved to an intrinsic, rather than financial worth, due to their exalted position as loved family members (54). The contract between vets and society may thus have altered such that the agreed professional ends of companion vets differ from vets in other sectors, and from the previous, utilitarian veterinary model.

To further complicate matters, in companion practice veterinarians must remain highly attuned to individual preferences as owners can report widely varying expectations regarding the role of vets in decision making (3, 55). Some owners expect vets to exert full Aesculapian authority and take decisions for them. An abdication of one's right to autonomy has been described as “voluntary diminished autonomy” in the human medical context (56) and these situations highlight the difficulty between a professional imperative to afford owners autonomy, and what many owners may want and expect of their vet. As owner autonomy remains in a position of the legal high ground, a veterinarian that accepts an owner's request for devolution of decision-making risks reprisal should the owner later wish a different outcome had occurred (33). Honestly answering the oft posed question “what do you think I should do” carries a degree of danger which is never more apparent than when grief and regret manifest litigiously. In some veterinary cultures this produces significant hesitancy to advocate or advise clearly on a preferred course of action, even when there is a direct request from the owner to do so. Beauchamp and Childress [(1), p. 108] answer critics of the “triumph of autonomy” by clarifying that although the principle engenders a right of an autonomous individual to choose, it does not confer a duty for them to do so. Understanding this distinction may perhaps allow for veterinarians to remain compliant yet cater to the owners who prefer to not uptake their autonomous rights.

Choosing treatment options may follow a “choice architecture” (50) approach as described by Yeates and Main (35) where options are curtailed or presented in such a way as to exert influence on clients toward the best outcome for the patient, or a Millian autonomy which suggests that customers ought to be provided with, and made free to choose from, all available options regardless of reasonableness of their choice (8). Under this reading of the principle, it is possible to justify a judgement free “vending machine” approach. Indeed, to enhance autonomy across healthcare sectors there is an expectation to offer choices (57) and many veterinary clients likewise highly regard their own ability to choose and expect the freedom to do so (35, 55). Practicing veterinary medicine in this way may be motivated by conforming to the choice zeitgeist, aversion to being labeled paternalistic, or avoidance of blame for negative case outcomes (55). While a vending machine approach affords owners complete decision-making autonomy, some authors question the ethics of practitioners deferring to owner decisions in this way (15, 41, 55, 58). Is it ethically acceptable for veterinarians to simply defer to owner decisions based solely on their autonomous rights, or could it be argued as an abnegation of a vet's duty to their patients?

In swinging toward autonomy, the pendulum of primacy swung away from benevolence, which could be argued as the guiding tenet of the much-maligned paternalistic approach. There has been limited, but compelling argument that this pendulum may have swung too far, and that owner autonomy has been afforded too great a credence and should be re-evaluated (8). We can't say for sure what society wants of vets—it would be a good exercise to investigate—but we can perhaps triangulate by considering occasions when the profession appears to deviate from it. There are increasing private and public allegations of clinicians pressuring owners toward more expensive treatments, sometimes in relation to whether animals are insured; supporting, or not doing enough to stop breeding for extreme features, or generally not seeming to have animal welfare at the core of their work (45, 59, 60). When vets are believed to diverge from protecting animal welfare and to be in pursuit of other goals (such as profit making or personal advancement) the tacit agreement of the social contract is jeopardized. Such accusations can be vehement and suggest that, for companion animals at least, upholding and advocating for animal welfare is implicit within the veterinary social contract and is the publicly agreed role of the profession.

Failure to uphold the social contract risks more than individual animal welfare compromise, it may raise questions of trust in the veterinary profession as a whole. While public interest in the welfare of companion animals continues to grow, the profession may lag behind changing social views and expectations in the companion sector. Coupled with a lack of regulatory evolution (54) the failure to have “courage, imagination and humility” (18) to adapt alongside evolving social ethics may threaten the trusted position of the veterinarian as upholder of animal welfare.

### Professional autonomy and moral injury

Owner autonomy can detract from a veterinarian's ability to exercise their professional autonomy over decisions. Professional autonomy is often thought of as relating to professional self-regulation, governing bodies etc. however, Thistlethwaite and Spencer [(61), p. 19] outline another aspect whereby members of a profession aspire to exercise autonomy over their *own decisions*, not in pursuit of self-interest, but to provide optimum service to the public interest. Wallace [(62), p. 292] defines autonomy in this sense as “refer(ing) to decision latitude or skill discretion that reflects control over one's own work and (as) an important resource in coping with job stress”. If we continue the argument that public interest is served by veterinarians seeking to optimize animal (patient) welfare, cases where patient benevolence and owner autonomy conflict, may not only impact on a vet's ethical responsibilities toward the proper ends of their profession [see Oakley and Cocking (63), Chapter 3 for relevant discussion], but also be detrimental to the veterinarian themselves (64).

Previous research illustrates how commonly vets feels they cannot “do the right thing” and are asked to provide futile treatments (17). Professional autonomy is not only threatened in these instances but can instrumentalise veterinarians to the extent that they are the physical agents of negative welfare states for their patients. The physicality and emotional impact of causing patients harm, against one's professional and moral views has not been widely explored in the veterinary profession [however, see Ashall (22) for methodological suggestions], but there is increasing interest in the role of moral distress, including the possible consequence of moral injury (65) on veterinary mental health. Crane et al. (66) define moral distress as “the experience of psychological distress that results from engaging in, or failing to prevent, decisions or behaviors that transgress, or come to transgress, personally held moral or ethical beliefs”. Burnout, which is often conflated with moral injury (65) has even been linked to a lack of professional autonomy (67).

While professional declarations may promote the primacy of a duty toward animals, in the absence of owner consent, vets have very few powers to protect animal welfare (41). Despite undeniable skill and artistry deployed by many vets in guiding owner decisions to converge with animals' best interests, vets remain constrained culturally and regulatorily, as well as by a legal system that leaves animal treatment up to the personal ethics of owners (68). Deferring to owner decisions based on their autonomous rights suggests an acceptance that a veterinarian's primary duty is to owners rather than animals, a position that may once have served societies expectation of the profession in an agricultural model of animal relationships, but as the companion animal sector has grown and human relationships with these animals becomes ever more relational, this model may no longer fit. Evolution of the social contract between public and the profession, consequent to a changing social ethic toward companion animals, leads to conflict between expectation of veterinary role as advocate for animal welfare, and the constraints posed by owner autonomy. This tension risks not only animal welfare and public trust in the profession but may even play a role in harms to veterinarians themselves by curtailing their professional autonomy.

## Discussion

Transposing the authority of autonomy to owners owes little to the social movement toward a respect for patient autonomy in human medicine. The latter is predicated on a direct relationship between patients and their doctor and affords patient self-determination over bodily integrity. The former protects owner rights of self-determination over property and has no bearing on protection of the patient. If we accept that the principle of respect for autonomy has been erroneously applied to owners in the veterinary sector, and that this misstep has serious consequences for both the integrity of the profession and animal patients, what ethical framework ought vets employ in cases where owner wishes deviate from a patient's interests?

Despite scant academic consideration of the difficulties caused by importing autonomy and informed consent to the veterinary sphere, those authors who have, advise a variety of alternative protocols. Veterinary clinical practice has more recently followed human medicine in promotion of collaborative, relationship centered care, which employs a shared decision-making framework, both of which have been identified as best practice (69). These concepts focus on communication between vet and client and promote an equal power balance and open exchanges of information and preferences, but don't explicitly require consideration of a patient's interests. In contrast, Gray et al. (8) advocate for a “middle way” where client autonomy is constrained to allow beneficence to the patient to take equal weight in cases where welfare concerns exist. Similarly, Hernandez et al. (55) advise that despite the power of the autonomy principle it is beholden upon veterinarians to “speak up” on behalf of patients. While not specifically considering the veterinary medical field, Cohen (50) also puts forward two alternative models where beneficence and autonomy rely upon each other rather than conflict; as such, decision making under these models requires each principle to give a little ground to the other, neatly dispensing with paternalistic concerns, as well as providing a framework to move forward with dilemmas.

A more challenging but illuminating paradigm I will explore here is that of best interests. Walker (24) highlighted the lack of mutual exclusivity between respect for autonomous decisions, and respect for autonomous individuals. This allows us to move away from accepting animal treatment decisions based solely on the rights of an autonomous owner. Instead, decisions about companion animal care may be transparently viewed as being made by proxy with incumbent standards applied, such as the requirement that a decision is in an animal's best interest. Indeed, some authors have drawn direct comparisons between companion animal healthcare, and the parent, child, pediatrician paradigm (19, 31) and others have suggested the standard of best interests be incorporated in veterinary ethical frameworks (70).

In human medicine, when a patient is incompetent to make an autonomous decision about their care (such as children and incapacitated individuals) decision-making passes to a proxy, such as a next of kin. In such cases it remains for the physician, who's sworn duty is solely to the welfare of the patient, to evaluate the quality of the surrogate's decision and intervene when appropriate (15, 71, 72). Ought this responsibility to evaluate and intervene be made more explicit in veterinary medicine? Rollin (68) states that (in his opinion), while over 90% of veterinarians are inclined toward a human pediatric model where beneficence to the patient is paramount, they remain thwarted by a respect for owner autonomy which is comparable to a motor mechanic model of veterinary practice. Furthermore, in their animal welfare strategy, the BVA make the case that veterinary patient best interests ought to be prioritized. This publication directly compares the veterinary relationship with that of pediatricians, and asserts that similarly, owner wishes, or veterinary career development should not be the focus of decision making [(49), p. 20]. If this “potentially radical” [(18), p. 254) argument is extended logically, it leads to the application of a standard of best interests.

Moving the companion veterinary medicine sector toward a position akin to pediatricians in evaluating what is in a patient's best interests would require two important resolutions: first, to agree a central tenet of the companion profession. The author proposes that this tenet be to maximize animal welfare. Second, to ensure greater understanding across the profession as to what good animal welfare is.

Human medics enjoy the simplicity that the sanctity and preservation of life provide a central rallying point around which decisions can be made (73, 74). Health professionals serving non-humans lack the luxury of such an uncomplicated centrality. Euthanasia and the ability to end suffering remain an important and socially desired function of the veterinary profession, as in the non-human arena, death is not considered a welfare concern [for debate on this point see Yeates (75)]. In comparison, prohibition against the taking of human life remains the very bedrock of human society. It is interesting to note however, both veterinary professionals and the public which they serve, are increasingly transposing the primacy of “life” to companion animals. Consequently, there is a growth in veterinary palliative care and in some quarters, belief that “natural death” of animals is to be exalted (76).

Competing priorities in veterinary care can obfuscate the central tenet of veterinary medicine. Several ethical decision-making tools have been developed to highlight how factors (such as owner and practitioner priorities) can gain primacy in decision-making and assist practitioners and owners to both acknowledge this is the case and allow focus on the animal patient (45, 77). Ever increasing skills, standards and possible treatments in the veterinary sector are of course to be welcomed, but without transparency as to the central tenet of veterinary care, it is lamentably easy for animal welfare to become secondary, tertiary or worse, to other concerns.

To address the second resolution regarding greater understanding of what good animal welfare is, a far greater focus should be applied to animal wellbeing, as opposed to “health”, in both undergraduate and continuing veterinary education. Commensurate focus ought be applied to ethology, species needs, animal psychology (and its consequence, behavior), welfare and quality of life assessment, as there is to disease diagnosis and treatment. While it is true that the best interests of animals are impossible for any human to know for sure, accuracy is surely improved by a deeper understanding of what it is like to be that animal, what brings it positive as well as negative emotional states, what matters to them, rather than to us.

Applying an animal focused rationalization to what course of action best protects and indeed, maximizes the positive welfare experience of individual animals also requires a nuanced consideration of their context and environment. Skipper et al. (46) put forward the concept of “contextualized care” which promotes the priority of patient welfare but allows for variation in decision-making based on the patient's situation, rather than a unidirectional best practice. Rather than stratifying animal healthcare by complexity of medical intervention, the ends of the profession may be better served by considering treatment decision-making as a dartboard, where maximized welfare within the circumstances represents the bullseye. We could conceptualize outer circles of the dart board as representing treatment decisions that prioritize non-patient factors such as providing futile treatments, delayed euthanasia, over or under treatment that negatively affect the welfare experience of the patient but are chosen due to owner or veterinary preference.

To change the view of treatment options from an intervention hierarchy to a concentric target with contextualized welfare at the center, requires a paradigm shift in the direction of travel of the companion sphere, where increasing specialization, pet insurance, undergraduate training, an increasingly litigious client base and widely publicized heroic treatments have tracked the profession in a direction of “gold standard” care. The idea of “gold standard” care is for some, a goal, for others a dangerous misnomer that creates the false impression of a hierarchy of treatment options, where the greater the intervention in magnitude, number or cost, the more gilded care the animal receives. That the profession itself is increasingly questioning this evolution, spurred on by concerns for both animal welfare and owners illustrates the need for re-evaluation.

## Conclusion

The unquestioned adoption of owner autonomy suggests a tacit acceptance that a veterinarian's primary duty is to owners rather than animals. Veterinarians remain impeded both by regulatory guidance and a legal system that leaves animal treatment up to the personal ethics of owners, despite professional declarations and oaths promoting the primacy of a duty toward animals. A changing social ethic may have altered the expectations that society has of those who provide healthcare for animals, resulting in a need to re-evaluate how autonomy is conceptualized, and informed consent utilized, in veterinary medicine. For the veterinary profession to uphold its end of the social contract and maintain a trusted position in society, it is essential to evolve the agreed ends of the profession to correspond with social morals toward companion animals as family members as opposed to property (78). This would be assisted by developing a clear moral purpose for the profession, supported by appropriate education and perhaps an ethical code of practice (79). Following in the footsteps of human medical ethics ignores the realities of the vet profession's more complex decision-making relationships and the profession should strive for the bravery and cohesiveness to forge a clear veterinary path. This paper considers the effect of reframing treatment decision-making; from autonomous owners giving informed consent, to a patient-centered approach with explicit acknowledgment that decisions are made by human proxies. It extends previous work to argue that a possible alternative framework to apply would be that of best interests, which would require clarification that the central tenet of the profession is to maximise animal welfare.

## Author contributions

The author confirms being the sole contributor of this work and has approved it for publication.

## Conflict of interest

The author declares that the research was conducted in the absence of any commercial or financial relationships that could be construed as a potential conflict of interest.

## Publisher's note

All claims expressed in this article are solely those of the authors and do not necessarily represent those of their affiliated organizations, or those of the publisher, the editors and the reviewers. Any product that may be evaluated in this article, or claim that may be made by its manufacturer, is not guaranteed or endorsed by the publisher.
